# Different expression of Defensin-B gene in the endometrium of mares of different age during the breeding season

**DOI:** 10.1186/s12917-019-2215-z

**Published:** 2019-12-21

**Authors:** M. Crociati, S. Capomaccio, M. T. Mandara, G. Stradaioli, L. Sylla, M. Monaci, K. Cappelli

**Affiliations:** 1 Department of Veterinary Medicine, University of Perugia, Via San Costanzo 4, 06126 Perugia, Italy; 2 Department of Agricultural, Food, Environmental and Animal Sciences, University of Udine, via delle Scienze 206, 33100 Udine, Italy; 3 “Centro di Ricerca del Cavallo Sportivo”, Department of Veterinary Medicine, University of Perugia, Via San Costanzo 4, 06126 Perugia, Italy; 4 “Centro di Ricerca di Medicina Perinatale e della Riproduzione”, Department of Surgical and Biomedical Sciences, University of Perugia, Piazza Lucio Severi 1, 06132 Perugia, Italy

**Keywords:** Endometrium, Gene expression, Defensin-β, Immune-modulation, Mare

## Abstract

**Background:**

Despite being one of the major causes of infertility in mares, the mechanisms responsible for equine endometrosis are still unclear and controversial. In the last few years, many investigations focused on local immune response modulation. Since it is generally accepted that endometrial fibrosis increases with age, we hypothesize that older mares could show altered local immune modulation, initiating a pro-inflammatory and tissue remodeling cascade of events that could lead to endometrosis. The aim of this study, indeed, is to evaluate and describe the local gene expression of genes involved in acute inflammatory response and fibrosis (*COL1A1, COL3A1, TNFA, MMP9, IL6, TGFB1* and *TGFBR1*), together with others associated to immune modulation (*DEFB4B, IDO1* and *FOXP3*), in uterine specimens from mares of different age.

**Results:**

Twenty-five Standardbred mares were involved in the study with age ranging from 7 to 19 years (mean 10.40 ± 4.42). They were divided by age into two groups: G1 (*n* = 15, less than 10 years old) and G2 (*N* = 10, greater than 11 years old). Specimens from the uterus’ right horn-body junction were collected and processed for histology evaluation and RT-qPCR assay.Gene expression of *DEFB4B*, *MMP9* and *TNFA* was higher in younger mares, suggesting a balance in immune modulation and tissue remodeling*. Interleukin-6* and *COL3A1* gene expressions were greater in older animals, probably indicating inflammatory pathways activation and fibrosis increase. Although no differences in fibrosis and inflammation distribution could be found with histological examination among G1 and G2, our results suggest a possible involvement of *DEF4BB* in regulating the local immune response in younger mare’s uterus (G1); age may contribute to the dis-regulation of *DEFB4B* transcription and, indirectly, influence the extracellular matrix homeostasis. Transcription of *IDO1* and *FOXP3* genes, instead, does not seem to be age related, or to be involved in local immune-response and tissue remodeling functions.

**Conclusions:**

Further investigations are needed in order to clarify the interactions between the expression of *DEFB4B, IL6*, *TNFA*, *COL3A1* and *MMP9* and other local signals of immune-modulation and tissue remodeling, in mares in a prospective study design.

## Background

Equine fertility is negatively affected by endometrosis, a progressive disease characterized by active or inactive periglandular and/or stromal endometrial fibrosis with subsequent morphological and functional mal-differentiation of endometrial cells [1–4].

Based on endometrial biopsy, Kenney [5] introduced histological classification to grade endometrosis in mares, which is considered a proxy of pregnancy maintaining probability. Despite this association, mechanisms responsible for endometrial modifications are still unclear [3, 6].

Raila [7] investigated the secretion rate of extracellular matrix components due to fibrosis in different degree of endometrosis; this Author described an altered collagen type I/III-ratio, while Walter et al. [8] reported no alterations in collagens I and III secretion. Studies based on immunohistochemistry and RT-qPCR revealed that the synthesis of pro-inflammatory mediators, such as interleukin-1α (IL1α), interleukin-1β (IL1β), interleukin-6 (IL6), interleukin-10 (IL10) and tumor necrosis factor-α (TNFA), alters the production of transforming growth factor-β (TGF-β), tissue inhibitor of metalloproteinase-1 (TIMP-1) and type-I collagen (COL1), thus inducing periglandular fibrosis [3, 4, 9, 10]. Equine species shows physiological breeding-induced endometritis, which is an acute phenomenon; due to this characteristics, some Authors suggested that repeated endometrial immune-challenge, acute inflammation and alteration of the endometrial healing process could increase the risk of chronic inflammation, facilitating collagen deposition and endometrosis [9–11].

We hypothesized that the balance between the activation of acute inflammatory pathways and the modulation of the local immune response could play a role in the development of endometrial fibrosis, by deranging the deposition of extracellular matrix components towards sclerosis. As representatives of the immune-modulation system, we focused our attention on β-defensins (*DEFB*), a new cluster of genes which expression has been characterized by Johnson et al. [12] in different tracts of equine female and male reproductive system. However, no correlations with grade and type of inflammation or with the degree of endometrosis are currently available. In regard to the modulation of immune response, interest has grown for Indoleamine 2,3-dioxygenase gene (*IDO*) and forkhead box P3 (*FOXP3*) genes. More in detail, IDO gene, regulates tryptophan synthesis both in bacteria and in cells. Immune modulation is exerted by controlling the proliferation of T regulatory (Treg) cells, T-lymphocyte subpopulation that controls the balance between immune activation and tolerance [13]. Treg cells play a role in the immune response through several mechanisms, for example inhibiting immune-regulatory cytokines such as TGF-β that, in turn, increase FOXP3 transcription factor expression [14]. *FOXP3* is expressed in T-regulatory lymphocytes during proliferation, and its increase is generally associated to an inflammatory response drop [15]. Moreover, in the presence of IL6, TGF-β promotes T-cell differentiation into T-helper-17 cells (Th17) and induces IDO. Since IDO is expressed by different cell types, including fibroblasts, both in case of inflammation and during tissue repair and remodeling [16], we hypothesized that IDO and FOXP3 could influence fibroblasts activation, chronic endometrial inflammation and subsequently fibrosis during endometrosis in the mare. In order to check these effects, we analyzed *IL6* and TNFA genes transcription, as inflammatory response representative molecules [3, 4, 9, 10]; transforming growth factor-β-1 (*TGFB1*) and transforming growth factor-β-receptor 1 (*TGFBR1)*, together with matrix metalloproteinase-9 (*MMP9*), as linked to tissue remodeling following the activation in pro-inflammatory pathways [3, 4, 9, 10, 17]. The expression of collagen type I-α1 (*COL1A1)* and collagen type III- α1 (*COL3A1)* as indicator of collagen deposition and progression towards fibrosis [7, 8].

Since it is generally accepted that endometrial fibrosis increases with age [5, 18, 19], we hypothesize that older mares could show altered local immune modulation, initiating a pro-inflammatory and tissue remodeling cascade of events that could eventually lead to endometrosis. Ricketts and Alonso [6] reported that animals with mean age of 9 years showed no signs of endometrial fibrosis, while others considered an average of 11 years as the line between healthy and first grade-affected mares [17, 18].

The aim of this study indeed is to evaluate and describe the local expression of genes involved in acute inflammatory response and fibrosis (*COL1A1, COL3A1, TNFA, MMP9, IL6, TGFB1* and *TGFBR1*), together with genes associated to immune modulation (Defensin-β 4B (*DEFB4B), IDO1* and *FOXP3*), in uterine specimens from mares of different age.

## Results

At the end of the breeding season, 10 out of 15 mares from G1 (66.7%) and 6 out of 10 mares in G2 (60.0%) were pregnant. Two out of 5 non pregnant mares from G1 were inseminated twice; after negative check, the Owner decided not to inseminate again. In G2, one mare had pregnancy loss at the end of the first semester and even if the animal was inseminated again, no new pregnancy was diagnosed. The remaining three were not pregnant at the end of the breeding season. All mares which were pregnant at the end of the breeding season had normal foaling.

### Histology evaluation

**Fig. 1:**
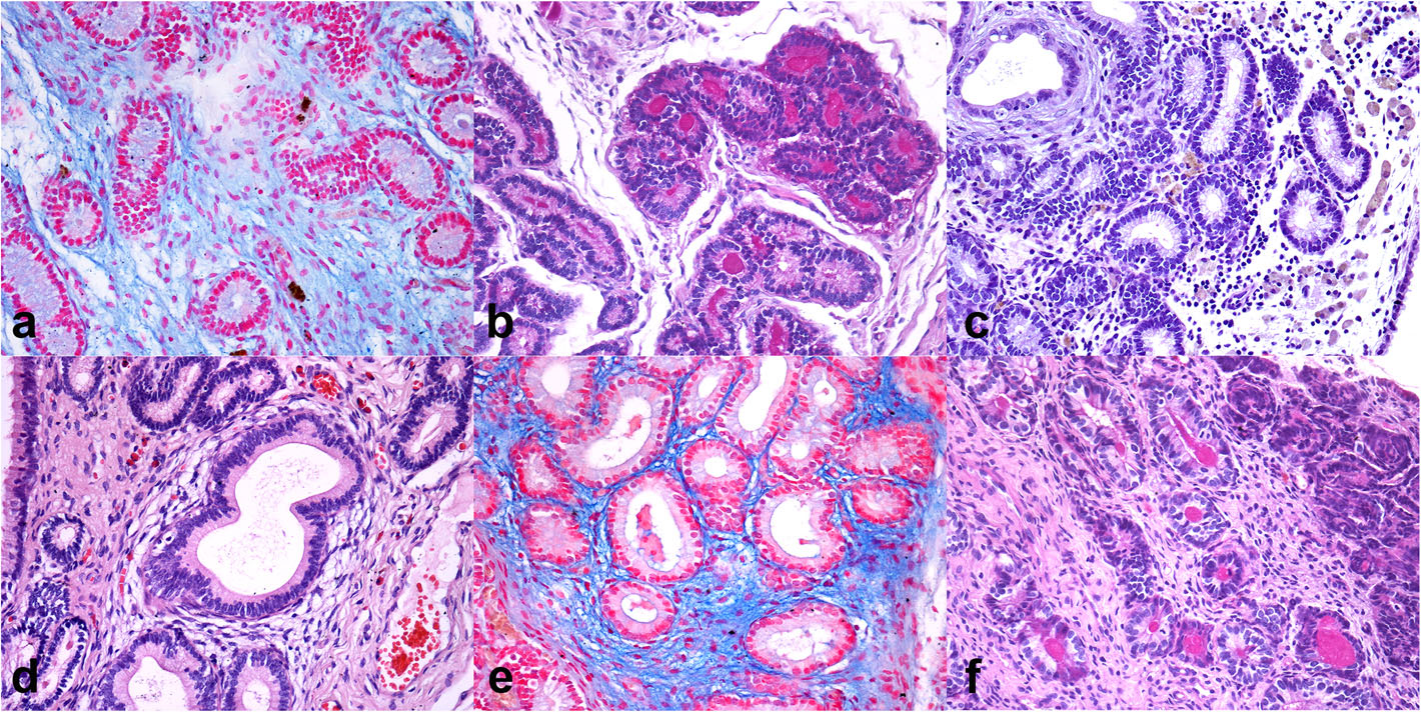
Histological findings of endometrial biopsies. (**A**-**C**) Group 1. (**A**) abundant mucopolysaccharide extracellular matrix (Alcian blue, 40x), (**B**) PAS-positive material in the glandular lumen (PAS, 40x); (**C**) Interstitial lymphomonocytic infiltration in the lamina propria of endometrium (H&E, 40x); (**D**-**F**) Group 2. (**D**) Endometriosis of grade II characterized by 4–10 cell layers thick fibrosis (H&E, 40x); (**E**) focal deposit of mucopolysaccharide extracellular matrix (Alcian blue, 40x); scattered glands showing intraluminal PAS-positive material in the glandular lumen (PAS, 40x)

In Fig. 1 histology findings of uterine biopsies, as representative of G1 and G2 are shown. In G1 mares (Fig. 1 a, b, c), periglandular fibrosis was observed in 13 cases, namely 9 and 4 cases of grades I and II of fibrosis, respectively. Fibrosis of grade III was totally absent. Periglandular fibrosis of grades I and II was associated to a mild glandular cystic dilation in 2 cases for each grade. The presence of Alcian blue-positive material in extracellular matrix was detected in 14 cases; for instance, mild (10 cases), moderate (3 cases) and abundant (1 cases). Periodic Acid-Shiff (PAS) positive material was detected in the glandular lumen in 8 cases, from mild (3 cases) to moderate (1 case) and abundant (4 cases).

Inflammation was observed in 10 out of 15 cases, that is mild (2 cases), to moderate (3 cases) and severe (5 case). In all the cases it consisted in lymphoplasmacytic infiltration (chronic form).

Uterine samples from mare of G2 group (Fig. 1 d, e, f) showed periglandular fibrosis in 8/10 cases, including grade I in 6 cases and grade II in the remaining 2 cases. Also in G2 fibrosis of grade III was not detected. Periglandular fibrosis of grades I and II was associated to a mild glandular cystic dilation in two cases. In addition, accumulation of Alcian blue-positive polysaccharide rich substances in extracellular matrix was detected in all cases from mild (5 cases), moderate (2 cases) to abundant (3 cases). Moreover, PAS-positive material was detected in the glandular lumen in 5 cases, from mild (1 case), moderate (3 cases) and abundant (1 case).

Inflammation was observed in 6/10 cases, from mild (1 case), to moderate (4 cases) and severe (1 case). It primarily consisted in lymphoplasmacytic infiltration (chronic form) occasionally associated with macrophages, except for a case of an acute mild eosinophilic infiltration (acute form).

### Gene expression

**Fig. 2:**
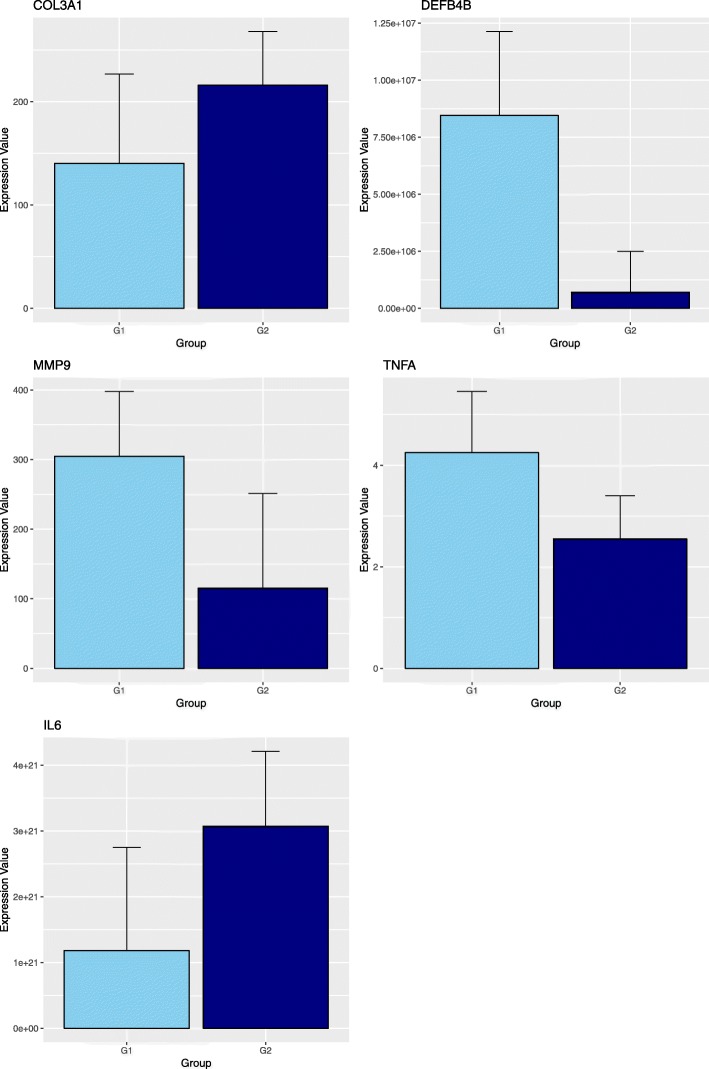
Barplot of the expression values between the two groups. G1: mares younger than 10 years old (*n* = 15); G2: mares older than 10 years old (*n* = 10). Differences were significant for COL3A1 and IL6 with *P* < 0.05, while expressions of MMP9 and TNFA were different with *P* < 0.01. Expression of DEFB4B was different with *P* < 0.001

**Fig. 3:**
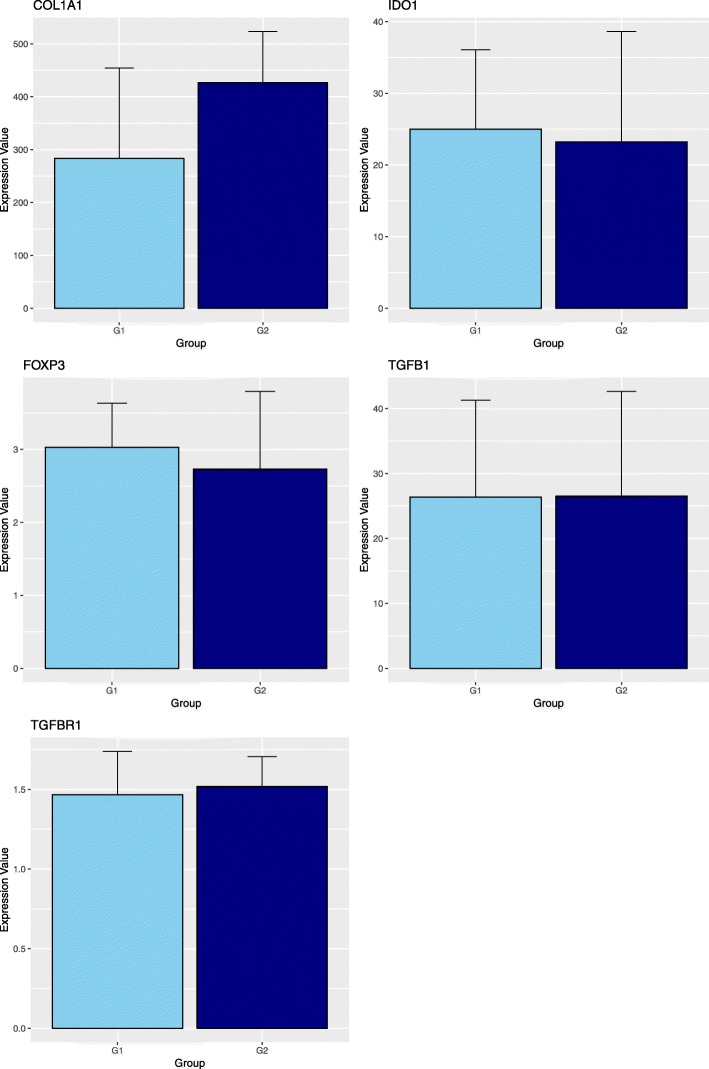
Barplot of the expression values between the two groups. G1: mares younger than 10 years old (*n* = 15); G2: mares older than 10 years old (*n* = 10). No significant differences were found between groups

Figures 2 and 3 show the relative expression distribution of *COL1A1*, *COL3A1*, *DEFB4B*, *FOXP3*, *IDO1*, *IL6*, *MMP9*, *TGFB1*, *TGFBR1* and *TNFA* transcripts of uterine biopsy of 25 horses grouped by age (younger and older than 10 years). The expression values are normalized with 3 reference genes: glyceraldehyde-3-phosphate dehydrogenase *(GAPDH),* Ribosomal protein L32 (*RPL32)* and beta-2-microglobulin *(B2M*). Reference genes showed relatively high stability with M value below the accepted threshold as stated by Vandesompele et al. [20]. Significant differences in gene expression between groups were found for *COL3A1*, *DEFB4B*, *IL6*, *MMP9* and *TNFA* (Fig. 2). More in detail, the expression of *COL3A1* and *IL6* is significantly higher in G2 animals, while *DEFB4B*, *MMP9* and *TNFA* transcription was greater in G1 mares. No significant differences were identified for other considered genes (Fig. 3).

## Discussion

Equine endometrosis is one of the major causes of endometrial mal-differentiation and embryo loss in mares. In this study, local gene expression relative to immune-modulation, inflammation and fibrosis markers has been analyzed in endometrial specimens belonging to mares of different age and with different fibrosis degree.

Our experimental mares were mainly classified as grade I and II of fibrosis, representing intermediate stages of endometrosis [5], with the exception of two mares per group, with no fibrosis at all. Groups were histologically homogenous and also inflammation severity showed equal distribution among animals; significant differences were represented by transcription of *COL3A1*, *DEFB4B*, *IL6*, *MMP9* and *TNFA* genes.

Recent studies hypothesize that modulation of the local immune response plays a role in the development of endometrial fibrosis [21]; a group of genes involved in the modulation of the immune response and in the activation of the innate immune-system are β-defensins (*DEFB*), which expression has been characterized by Johnson et al. [12] in equine reproductive tract but independently on age or endometrosis degree. In their study, Marth et al. [22, 23], found higher *DEFB* transcription in mares susceptible to persistent post-breeding endometritis and the Authors considered this antimicrobial peptides as a marker of susceptibility. Correlation with the degree of endometrosis or age has not been investigated. At the best of our knowledge, this is the first report concerning *DEFB* gene expression in equine endometrium in relation to the age. More in detail, in the present study we found greater *DEFB* expression in G1 mares, that is in younger animals. Defensins are usually thought as antibacterial proteins, but recently their role in immune modulation and host-microbiome homeostasis has been advanced [24]. Since no antigenic challenge was induced in this study, we hypothesized that younger mares expressed *DEFB* at uterine level, as indicative of balanced local immune-modulation, thus resulting in reduction of inflammatory pathways and in slower progression towards fibrotic tissue deposition. This is supported by results concerning *IL-6* and *COL3A1*, which were more expressed in older animals (G2), while *MMP9* transcript was higher in G1, as discussed below. Hoffman et al. [1] suggest that epithelial damage in endometrial glands is the initiating event for endometrosis; damaged epithelial cells also show alteration in secretion of immune-modulatory molecules such IDO products and beta-defensins [25]; however, it is not clear how age could influence the activation of inflammatory and tissue remodeling pathways after glandular epithelial damage.

Our experimental groups are similar in histology scoring of fibrosis and local inflammation. It is possible that different expression of β-Defensins between younger and older mares could play a role in reducing the susceptibility to persistent endometritis and, indirectly, to fibrosis progression, which positively correlated with age in terms of severity. In order to confirm this hypothesis, experimental mares should be re-evaluated during following breeding-seasons; no data concerning the follow-up are available yet.

Transcription of *IDO* and *FOXP3* have been mostly investigated in case of inflammatory stimulation in equine species [15, 16, 26]; while information in relation to the age or the degree of endometrosis is still poor. In the present investigation, no differences in transcription for *IDO1* and *FOXP3* genes were identified in younger and older mares, and this is partly in agreement with Schöniger et al. [21], who found no difference in *IDO* transcription in both healthy and diseased equine endometrial specimens. We hypothesized that the low transcription of *IDO* gene could confirm the lack of bacterial challenge in the endometrium of mares involved in this study, since *IDO* pathway is reported to be activated during bacterial invasion [16]. *FOXP3* gene is usually activated by T-regulatory lymphocytes, including virus-induced proliferative lesions [15, 27]. Moreover, *IDO* and *FOXP3* transcription could be enhanced by TGF-β, which expression is reduced both in G1 and in G2 animals [14]. Since no variation in *FOXP3* and *IDO1* expression could be recognized in this study, it is likely that T-regulatory cells are not involved in tissue remodeling and collagen deposition. Some investigations focused on interleukin gene expression in experimentally induced endometritis [9, 10], or during the different stages of the estrous cycle [3]. Differences in biopsy collection timing or in experimental design made the comparison of our results sometime difficult. However, these studies reported an increased expression of IL1β, interleukin-8 (IL-8) and interleukin-1 receptor-α (IL1RA) in mares susceptible to persistent endometritis and a decreased expression of IL10, which is responsible for ending the local immune reaction. In the present study we focused on IL6, which is responsible both for initial stages of inflammatory response and for induction of collagen deposition, as in human species [3, 9]. Local expression of IL6 in our study was lower in G1 than in G2 mares, thus suggesting, together with the different expression of DEFB4B and MMP9, a balanced in immune-response and maintenance of extracellular matrix homeostasis. Our results are partly distinct from Christoffersen et al. [9] who found that mares resistant to persistent endometritis showed higher expression of IL6 after experimentally induced *E. coli* endometrial challenge, suggesting that IL6 could play a role in reducing bacterial invasion into the uterus. However, the aim of this study was not concerned with the evaluation of the susceptibility to persistent endometritis after an experimental immune challenge.

In a recent investigation by Szòstek et al. [11], IL6 challenge decreased the expression of MMP9 in equine endometrial explants cultured in vitro, only in Kenney I and IIA classes, while the opposite result was observed in IIB and III Kenney classes. Gene expression of IL6 and MMP9 in our specimens was in accordance with those results, since we found increased expression of the IL6 together with reduced transcription of MMP9 in older animals (G2), when compared to G1 mares. MMP9 indeed is involved in tissue remodeling and collagen fibers degradation. This gene is particularly expressed in equine endometrium glandular and periglandular stromal cells [28]. In the study reported by Aresu at al [28]. however, there was no difference in the expression of MMP9 in younger and older mares; this seems not in agreement with our results but we identify at least two explanations. First, immunohistochemistry and gene expression assays are not directly comparable due to post-transcriptional regulation issues. Second, differences in the trend of tissue remodeling in the two experimental cohorts may exist: histology investigations did not show any sign of active inflammation in our G2 mares. This can negatively influence the expression of MMP9, which is probably more linked to inflammation rather than fibrosis [28].

Fielding et al. [29] anyway, tried to explain how repeat acute resolving inflammation drives tissue damage combining the effect of IL6 and metalloproteinases (MMPs): repeated inflammation induces IL6-mediated T helper 1 and the Signal transducer and activator of transcription-1 (STAT1) pathway signaling activation, leading to extracellular matrix turnover disruption by MMPs activity.

Concerning tissue remodeling and collagen deposition, we investigated also the expression of *COL1A1* and *COL3A1*, together with *TGFB1* and *TGBR1*; only *COL3A1* showed significantly increased expression in G2 animals. In a recent investigation Szòstek et al. [30] evaluated the effect of *TGFB1* on in vitro cultured endometrial fibroblast and observed an increase in the expression of alpha-smooth muscle actin (α-SMA), COL1A1 and COL3A1, as indicative of myofibroblast activation and extracellular matrix component deposition. Although we found greater expression of *COL3A1* in our G2 mares, we could not confirm those results. This divergence could be due to differences in signaling and regulatory pathways or in the bioavailability of interleukins in tissues examined ex vivo or cultured in vitro*.*

The cytokine TNFA is considered to be indicative of acute inflammatory process but it is also involved in the regulation of equine estrus cycle [31, 32]. In their study, Centeno et al. [31] found any differences in *TNFA* expression in endometrial tissue of mares with different degree of fibrosis; the expression of *TNFA* in our study was greater in G1 mares than older ones. It is possible that the stage of the estrous cycle at the moment of the uterine biopsy influenced the *TNFA* expression in specimens: in fact, Centeno et al. [31] used tissues deriving from mare in diestrus (D_5_ – D_10_), while in the present study, biopsies were performed during oestrus.

Percentage of pregnant mares at the end of the breeding season was similar (66% in G1 and 60% in G2, respectively). The small number of mares limited the possibility of deeper investigation concerning influence of gene expression on percentage of pregnant mares, as the number of groups that could be made. In fact any attempt to create other age-groups failed to evidence further statistical differences. However, different transcription of *DEFB4B* in older compared to younger mares points out possible variation in uterine environment in relation to the age in equine species.

Although no differences in fibrosis and inflammation classes’ distribution among groups could be found at histology examination, our results suggest possible involvement of *DEFB4B* in regulation of the local immune response into the uterus of younger mares; age progression could contribute to the dis-regulation of *DEFB4B* transcription and, indirectly, influence the extracellular matrix homeostasis.

Transcription of *IDO1* and *FOXP3* genes, instead, seems not to be influenced by the age, nor to be involved in local immune-response and tissue remodeling functions. However, deeper investigation is needed to clarify the interactions between the expression of *DEFB4B, IL6*, *TNFA*, *COL3A1* and *MMP9* and other local signals of immune-modulation and tissue remodeling, in mares in a prospective study design.

## Conclusions

In the present study, the gene transcription of immune modulation genes *DEFB4B*, *FOXP3* and *IDO1*, together with genes involved in acute inflammatory response and fibrosis (*COL1A1, COL3A1, TNFA, MMP9, IL6, TGFB1* and *TGFBR1*) was evaluated for the first time in endometrial specimens from young compared to older mares. Although no differences in fibrosis and inflammation classes’ distribution among groups could be found at histology, our results suggest possible involvement of *DEFB4B* in regulation of the local immune response into the uterus of younger mares and, indirectly, its influence on the extracellular matrix homeostasis. Transcription of *IDO1* and *FOXP3* genes, instead, seems not to be influenced by the age, nor to be involved in local immune-response and tissue remodeling functions. However, further investigations are needed to clarify the interactions between the expression of *DEFB4B, IL6*, *TNFA*, *COL3A1* and *MMP9* and other local signals of immune-modulation and tissue remodeling, in mares in a prospective study design.

## Methods

### Animals and specimen collection

A total of 25 Standardbred multiparous mares were involved in the study. Age of the mares ranged from 7 to 19 years (mean 10.40 ± 4.42 st.dev.) while parity ranged from 2 to 10 (mean 4.6 ± 2.08 st.dev.). Animals were from different breeding centers, but all of them were under the reproductive management of the same Practitioner. All experimental procedures and the care of the animals complied with the Italian legislation on animal care (Legislative decree n.26, 03/03/2014) and adhered to the internal rules of University of Perugia. The approval for conducting this study was also granted by the Practitioner responsible of breeding centers without a request to ethical committee because the samples were collected as part of breeding soundness evaluation of mares. The owners of the mares gave us written consent to use the data freely for research purpose.

All the subjects were first evaluated in order to declare them clinically healthy; then, a complete breeding soundness examination was performed to identify the estrus (uterine edema, lumen accumulation of fluid, preovulatory follicle of at least 3.5 cm in diameter), which was designated as the time suitable for the execution of uterine biopsies. The genital tract was evaluated by ultrasound device with 5.0–8.0 MHz linear transrectal transducer (Mindray M7Vet, Mindray Medical Italy Service srl). Mares with systemic signs of illness, positive bacterial culture, or which showed spotted uterine fluid accumulation and/or purulent vaginal discharge, were excluded from the experimental design.

Uterine biopsies were routinely executed at stud farm by the Practitioner in order to evaluate endometrial wall at the beginning of every breeding season. Specimens were collected and sent to the Department of Veterinary Medicine, University of Perugia, Italy, for histology evaluation. In accordance with the Practitioner, for this experiment an aliquot of each biopsy was used also for gene expression evaluation. Surgical scrub of vulva and perineal region was performed with 7.5% Povidone-Iodine solution (Betadin® Meda Pharma S.p.A. Milano, Italy); then an alligator-type biopsy forceps (Equivet®, Krusee, Marslev, Denmark) was introduced trans-vaginally with a gloved hand through the cervix. Once reached the uterine lumen, the gloved hand was inserted into the rectum and pulled a portion of uterine wall into the branches of the forceps. A specimen of tissue from the right horn-body junction was collected and divided into two portions, each one averaging 0.5 × 0.5 × 0.3 mm on size. One portion was immediately fixed in buffered 10% formalin solution in an empty tube and stored at room temperature for 24 h, before fixation and routine histopathological evaluation. The second fragment was immediately put into empty cryovials tubes, snap-frozen in liquid nitrogen and stored at − 80 °C until gene transcription analysis.

### Histology procedure

After fixation samples addressed to histological evaluation were dehydrated and paraffin embedded. Five μm Formalin-Fixed Paraffin-Embedded (FFPE) sections were routinely stained with Hematoxylin and Eosin (H&E). Staining with Periodic Acid-Shiff (PAS) and Alcian Blue were also performed to highlight polysaccharide rich substances in the glandular lumens or extracellular matrix. Moreover, staining with Masson’s trichrome was used to aid in detection of fibrosis. For each biopsy sample fibrosis and inflammation were assessed in details. Based on number of periglandular spindle cell layers, fibrosis was graded as mild (grade I: 1–3 cell layers), moderate (grade II: 4–10 layers) and severe (grade III: more than 11 layers) [5]. As for inflammation, the assessed parameters included the leucocyte cells (lymphocytes, neutrophils, macrophages), the severity (focal = mild, multifocal = moderate, diffuse = severe) and duration of the process (acute, chronic).

### RT-qPCR

For each sample, total RNA was extracted from 100 mg of ground tissue using the Trizol Plus RNA purification kit (Ambion, Life Technologies, Monza, Italy), according to the manufacturer’s instructions. The RNA concentration was assessed using the NanoDrop1000 spectrophotometer and the integrity of RNA was examined by electrophoresis in a denaturing 1% agarose gel.

Total RNA (500 ng) of each sample was reverse transcribed using the SuperScript® VILO™ Master Mix (Thermofisher), according to the manufacturer’s recommendations. Primers on reference genes (*GAPDH, RPL32* and *B2M*) were taken from previous investigations [33, 34], while genes of interest (*COL1A1*, *COL3A1*, *DEFB4B*, *FOXP3*, *IDO1*, *IL6*, *MMP9*, *TGFB1*, *TGFBR1* and *TNFA*) were designed based on available sequences using the Primer-BLAST software [35], trying to locate them in different exons or at an exon–exon junctions to avoid biases due to genomic DNA amplification. Primer sequences and accession numbers for tested genes are listed in the Additional file 1: Table S1.

The RT-qPCR reactions were carried out aliquoting 5 μL of a ten-fold diluted cDNA and SYBR Select MasterMix for CFX (Thermofisher). The amplification was performed in a CFX96 Touch instrument (BioRad, Hercules, CA) with the following conditions: 98 °C for 3 min, then 40 cycles of 98 °C for 10 s and 60 °C for 15 s. Fluorescence data were collected at the end of the second step and, following cycling, the melting curve was determined in the range of 58–95 °C with an increment of 0.01 °C/sec. Each reaction was run in triplicate with appropriate negative controls.

Raw data analysis was carried out with Bio-Rad CFX Manager software (ver. 3.1.157). To analyze gene expression stability of HKGs, geNorm algorithm, included on CFX Manager software was applied [20]. The ΔΔCq method was applied in GeneEx Pro (ver. 6.0) accounting for reference genes expression and setting to 1 the lowest expressed sample for each gene. Calculated relative expression values for genes of interest were then imported into RStudio software (v.3.2) [36] for statistical analysis.

### Statistical analysis

According to the hypothesis that age could influence the expression of local immune-modulator genes, samples were divided in two groups: 15 mares in Group 1 (younger than 10 years, G1) and 10 mares in Group 2 (older than 10 years, G2). When appropriate, data were expressed as mean ± st. dev. Variables other than age, that is fibrosis degree, parity and barren years were evaluated for correlation with gene expression but none resulted to be significant except age and fibrosis. To evaluate differences between the two groups, a linear model was used accounting also for the fibrosis status that was set as the second response variable. Analysis of variance (ANOVA) was applied to verify statistical significance (*p*-value < 0.05).

## Supplementary information

**Additional file 1: Table S1.** Primer combinations and accession numbers for tested genes

## Data Availability

The datasets used and/or analyzed during the current study are available from the corresponding author on reasonable request.

## Abbreviations

α-SMA: Alpha-smooth muscle actin
B2M: Beta-2-microglobulin
COL1: Collagen I
COL1A1: Collagen I type-α1
COL3A1: Collagen III type-α1
DEFB: Defensin-β
DEFB4B: Defensin-β 4B
FFPE: Formalin-Fixed Paraffin-Embedded
FOXP3: Forkhead box P3
G1: Group 1
G2: Group 2
GAPDH: Glyceraldehyde-3-phosphate dehydrogenase
H&E: Hematoxylin and Eosin
IDO: Indoleamine 2,3-dioxygenase
IDO1: Indoleamine 2,3-dioxygenase 1
IL10: Interleukin-10
IL1RA: Interleukin-1 receptor-α
IL1α: Interleukin-1α
IL1β: Interleukin-1β
IL6: Interleukin-6
MMP9: Matrix metalloproteinase-9
MMPs: Matrix metalloproteinases
PAS: Periodic Acid-Shiff
RPL32: Ribosomal protein L32
STAT1: Signal transducer and activator of transcription-1
TGFBR1: Transforming growth factor-β receptor-1
TGFB1: Transforming growth factor-β-1
TGF-β: Transforming growth factor-β
Th17: T-helper 17 cells
TIMP-1: Tissue inhibitor of metalloproteinase-1
TNFA: Tumor necrosis factor-α
Treg: T-regulatory cells
