# Supplementary material for: Different expression of Defensin-B gene in the endometrium of mares of different age during the breeding season
Source: BMC Vet Res. 2019 Dec 21;15:465. doi: 10.1186/s12917-019-2215-z (PMC6925900; doi:10.1186/s12917-019-2215-z)
Supplement: Supplementary file 1 — Additional file 1: Table S1. Primer combinations and accession numbers for tested genes [file 12917_2019_2215_MOESM1_ESM.docx]

| Gene | Accession number / Reference | Primer Forward | Primer Reverse |
| --- | --- | --- | --- |
| *COL1A1* | XM_023652710.1 | CAAGAGGAGGGCCAAGAAGA | GGTTTCCATACGGCTCGGTC |
| *COL3A1* | NM_009930.2 | CCTCATTGTCTTCCTGTTGCCT | GTGCCGATCTGTTTTGACCCT |
| *FOXP3* | NM_001163272.1 | CCTATGCCACTCTCATCCGC | CTGAAGAAGGCGAACATGCG |
| *IDO1* | XM_014736538.2 | CTTCTTGTCTACGCAACGCC | ACGCCTTCATAGAGCAGACC |
| *TGFBR1* | XM_023629741.1 | CCGTTTGTATGTGCACCGTC | CCAAGGCCAGATGATGGCT |
| *DEFB4B* | NM_001081887.1 | CCTCATTGTCTTCCTGTTGCCT | GTGCCGATCTGTTTTGACCCT |
| *TNFA* | (Mignot et al. 2012) | AGCCTCTTCTCCTTCCTCCTT | CAGAGGGTTGATTGACTGGAA |
